# Understanding the Nutritional Needs of Transgender and Gender-Nonconforming Students at a Large Public Midwestern University

**DOI:** 10.1089/trgh.2019.0071

**Published:** 2020-03-16

**Authors:** Sierra R. Kirby, Jennifer A. Linde

**Affiliations:** ^1^ Desert Farming Initiative, College of Agriculture Biotechnology and Natural Resources, University of Nevada, Reno, Reno, Nevada.; ^2^ Division of Epidemiology and Community Health, School of Public Health, University of Minnesota-Twin Cities, Minneapolis, Minnesota.

**Keywords:** transgender, gender nonconforming, students, nutrition, health

## Abstract

**Purpose:** The purpose of this research was to generate knowledge of the nutrition-related health disparities and barriers to adequate nutrition and health that transgender and gender-nonconforming (GNC) university students experience.

**Methods:** A needs assessment was conducted with 26 transgender/GNC students enrolled at a large public Midwestern university from November 2017 through February 2018. Interviews and surveys were used to collect qualitative and quantitative data regarding nutrition-related health disparities and barriers, and to identify potential interventions to reduce the disparities or barriers.

**Results:** Major themes emerged around food insecurity, body image, nutrition knowledge and skills, dietary intake, and barriers to healthy eating and positive body image. Other themes included inclusiveness of existing resources and resource needs and preferences.

**Conclusion:** Participants identified interventions that could be introduced on campus to improve their health and nutrition status. Comparisons between study participants and LGBTQ (lesbian, gay, bisexual, transgender, queer) populations in the Midwest and with the general student population at the same university show poorer results for dietary intake, body image behaviors, and food security among transgender/GNC study participants. Overall, this study provides a deeper understanding of nutrition-related needs among transgender/GNC university students.

## Introduction

Transgender and gender-nonconforming (GNC) communities are vulnerable populations characterized by the disadvantaged distribution of social goods and services that affect health outcomes.^1^ A review of existing literature illuminates the lack of information available on LGBTQ (lesbian, gay, bisexual, transgender, queer) health, and transgender/GNC communities in particular. Of the research on transgender health that is available, most work focuses on sexually transmitted diseases, especially HIV/AIDS.^2^

However, some research is available regarding nutrition-related health disparities that affect transgender/GNC communities, including eating disorders, food insecurity, and chronic diseases.^3–6^ For example, studies have found that transgender communities are at a higher risk of cardiovascular disease and myocardial infarctions when compared with their cisgender counterparts.^4,5^ However, more research is needed to better understand how these conditions are related to factors such as hormone treatment and dietary habits in transgender/GNC populations. Previous studies, including one focusing specifically on university students, confirmed that transgender participants experience significantly increased odds of disordered eating behaviors compared with cisgender counterparts. Evidence supports disordered eating behaviors among transgender individuals as a way of suppressing or accentuating gender identifying features largely by means of weight and body size changes provoked by restrictive or compensatory eating disorder behaviors.^7,8^ Previous research has found that 35.4% of transgender respondents reported food insecurity, or a lack of reliable access to affordable food of sufficient quantity and nutritional quality, over a 12-month period.^4^ Food security varied by age, with 18- to 24-year-olds reporting higher rates of food insecurity than other age groups.^4^ Although specific research on transgender and GNC college students is not available, in general, college students experience food insecurity at significantly higher rates than the general U.S. population.^9^

Overall, addressing health disparities among this population is important because health and well-being have been associated with academic success; and research has found that completing higher education has been linked to improved health and socioeconomic opportunity.^4,10^ For example, adults without a high school diploma by age 25 in the United States are likely to die 9 years earlier than college graduates.^10^ Educational attainment is a particularly critical issue for transgender/GNC communities that face many barriers to education early on.^5,11,12^ For example, a 2015 survey conducted by the National Center for Transgender Equality found that 17% of transgender respondents left their K-12 school and 2% left their college or vocational school due to mistreatment.^11^ In addition, studies done in LGBTQ communities in the Midwest have found that increased access to education and employment can lead to increased food security, decreased homelessness, and the ability to afford health care costs.^4^

Few data are available on nutrition-related health disparities and barriers to adequate nutrition and maintaining health that are experienced by transgender/GNC university students. Consequently, the purpose of this research was to explore this gap in knowledge within a large public university in the Midwestern United States. This study focuses on two questions: (1) What are the nutrition-related needs and barriers to health among transgender/GNC students on campus, and (2) what are potential interventions that could be introduced on campus to improve the health and nutrition status of transgender/GNC students?

## Methods

Students who identified as transgender or GNC, were enrolled at the flagship campus of a large public Midwestern University, were ∼18 years old, and were able to read and speak English were eligible for this study. All study procedures were approved by the University's Institutional Review Board (IRB). Community and university organizations were used to recruit students through flyers, email advertisements, and communication from leaders. Emails were sent to community and university organizations that work with transgender/GNC students to obtain permission to post tear-off flyers advertising the research study in their building or office, for an advertisement for the research study to be incorporated into their email listserv newsletters, and/or for organizational leaders to inform students about the research study through word of mouth. Participants were recruited from November 2017 through February 2018.

The study procedures consisted of two options. The first option was a one-time, in-person interview with the researcher. The interview included a premeeting screening email to ensure study eligibility. During the interview, the participant was given an anonymous written questionnaire with questions relating to demographics, general health, and health habits. The researcher also asked open-ended questions. The second option was a one-time, anonymous online survey with the same questions as the in-person option, as well as space to respond to open-ended questions. The survey option was included to increase participation by providing convenience and comfort when answering personal questions and reducing the potential risk of gender identity being revealed. Item content was identical between interview and survey formats.

All interviews were conducted by the researcher in a private, neutral location on campus. All interviews and surveys were presented in English, the primary language of instruction on campus. All participants signed a consent form. Audio recording was used to ensure accurate interview transcription. For survey participants, a link to a Qualtrics online survey was provided. Interviews and surveys took an hour or less to complete. After study completion, $10 USD gift card incentives were emailed to participants.

### Measures

#### Demographics

Questions regarding height, weight, and year in school were derived from the University's College Student Health Survey (CSS).^13^ Questions regarding age, sexual orientation, gender identity, sex assigned at birth, racial/ethnic background, employment status, and annual income were derived from Rainbow Health Initiative's Voices of Health (VHS) survey.^4^ The question about pre-existing medical conditions was adapted from the CSS and VHS items.^4,13^ Demographic questions regarding pronoun use, financial support, and transitioning status were created for this study.

#### General health and health habits

Questions regarding fruit, vegetable, and sugar sweetened beverage intake and regarding weight loss attempts, weight loss methods, and binge eating were derived from the CSS.^13^ Food insecurity questions were derived from the VHS.^4^ Body image questions were taken from a measure developed and validated in a prior study of transgender youth and young adults.^3^ Questions regarding resource utilization and about whole grain intake were added to this study.

#### Open-ended questions

Open-ended questions focused on information regarding barriers to eating fruits and vegetables, healthy eating, and positive body image as well as resource needs, health concerns, potential program logistics, and feedback and suggestions for the research team. All open-ended questions were developed for the study, except for the question regarding top 3 health concerns, which was derived from the VHS (Nezhad S and Flunker D, unpublished data, 2016).

### Data analysis

Twenty-eight students were recruited for the study. However, only data collected from 26 student participants were included in the analysis. Data from two participants were not included because one participant was not able to provide verification of student status, and the other did not complete any of the survey or open-ended questions. One student was interviewed in person. Their interview was recorded and subsequently transcribed. Qualitative data were analyzed using a transcript-based systematic thematic analysis to determine commonalities and divergences between information provided by interviewees.^14,15^ Interview transcripts were visually inspected, and the constant comparative method was used to reduce correlations and patterns into themes for interpretation.^16–18^ Qualitative analysis concepts such as extensiveness (the number of participants that mentioned the topic), frequency (the number of times a topic was mentioned), and specificity (the provision of specific details from experience rather than vague and impersonal responses), and content relevance were used to identify major and minor themes.^14,15,19^ Major themes were defined by extensiveness, specificity, content relevance, and overall key findings. Minor themes were defined by having less extensiveness, specificity, and emerged as subthemes to elaborate on overall key findings.^19^ Quantitative data from survey questions were analyzed by calculating means and frequencies. Quantitative results were compared with qualitative findings to further explain themes that emerged.

## Results

Table 1 displays participant demographic and background information. The average participant was 22.7 years old and had an average BMI of 24.9 kg/m^2^. The following qualitative and quantitative results have been divided into three subcategories, including dietary intake and food insecurity, body image, and resource utilization and needs.

**Table 1.** Demographic and Background Information of Student Participants (*n*=26)

	N	%
Year in school		
First-year undergraduate	7	27
Second-year undergraduate	7	27
Third-year undergraduate	2	7.7
Fourth-year undergraduate	1	3.8
Fifth year or more undergraduate	1	3.8
Master's degree	1	3.8
Doctoral or professional degree	7	27
Sexual orientation		
Lesbian	1	3.8
Gay	3	12
Bisexual	4	15
Pansexual	5	19
Asexual	1	3.8
Queer	12	46
Straight/heterosexual	0	0
Gender identity		
Female	0	0
Male	2	7.7
Transfemale/transwoman	1	3.8
Transmale/transman	7	27
Genderqueer/Gender nonconforming	6	23
Written-in different identity (genderfluid, neutrois, agender, nonbinary guy, nonbinary, nonbinary/agender, demigender, transgender nonbinary, nonbinary transman)	10	38
Sex assigned at birth		
Male	1	3.8
Female	25	96
Intersex	0	0
Race, ethnicity		
Black/African American	1	3.8
White/Caucasian	22	85
Hispanic or Latino	4	15
American Indian/Alaskan Native	1	3.8
Asian or Pacific Islander	2	7.7
Employment status		
Full-time	1	3.8
Part-time	18	69
Unemployed	7	30
Income		
> $10,000	9	35
≤ $10,000	10	38
Unanswered	7	27
Receives familial financial support	16	62
BMI		
Underweight (<18.5)	1	3.8
Healthy weight (18.5–25)	18	69
Overweight (25–30)	3	12
Obese (>30)	4	15
Gender-affirming medical interventions	13	50
Hormone replacement therapy	7	27
Gender-affirming surgery (e.g., “Top Surgery,” “Tubal Ligation”)	5	19
Intended hormone replacement therapy	1	3.8
Intended gender-affirming surgery	4	15
Medical conditions		
Anxiety	22	85
Depression	20	77
Anorexia	2	7.7
Bulimia	5	19
Other eating disorders (ARED, ARFID, EDNOS, binging addiction/reliance)	4	15
Obesity	1	3.8
None of the above	3	12

ARED, avoidant/restrictive eating disorder; ARFID, avoidant/restrictive food intake disorder; EDNOS, eating disorder not otherwise specified.

### Dietary intake and food insecurity

Table 2 displays the total number and percentage of food participants consumed over a 7-day period. Over 50% of participants did not drink 100% fruit juice, diet soda, sweetened drinks, or coffee drinks with added sugar. This is important to note because limiting the consumption of these food items is recommended.^20,21^ Approximately 46% of participants did not eat fruit daily during the 7-day period, which is below the recommendation of two cups of fruit per day.^22^ Forty-two percent did not eat vegetables daily during the 7-day period, which is below the recommendation of 2.5–3 cups of vegetables per day.^23^ Finally, 58% of participants did not eat whole grain food products daily during the 7-day period, which falls below consumption recommendations of three 4-oz equivalents of whole grain foods daily.^24^

**Table 2.** Student Participant Dietary Intake Over a 7-Day Period (*n*=26)

	Did not eat or drink, N (%)	1–3 times, N (%)	4–6, times N (%)	1 time per day, N (%)	2 times per day, N (%)	3 times per day, N (%)	≥4 times per day, N (%)
100% fruit juice	14 (54)	6 (23)	3 (12)	1 (3.8)	0 (0)	0 (0)	2 (7.7)
Fruit	3 (12)	8 (31)	1 (3.8)	6 (23)	6 (23)	2 (7.7)	0 (0)
Vegetables	1 (3.8)	6 (23)	4 (15)	4 (15)	8 (31)	2 (7.7)	1 (3.8)
Soda or pop	12 (46)	6 (23)	4 (15)	2 (7.7)	0 (0)	2 (7.7)	0 (0)
Diet soda or pop	23 (88)	2 (7.7)	0 (0)	0 (0)	0 (0)	0 (0)	1 (3.8)
Sweetened drinks	17 (65)	6 (23)	1 (3.8)	1 (3.8)	1 (3.8)	0 (0)	0 (0)
Coffee drinks with added sugars	15 (58)	5 (19)	0 (0)	3 (12)	2 (7.7)	1 (3.8)	0 (0)
Whole grain foods	6 (23)	5 (19)	4 (15)	5 (19)	3 (12)	3 (12)	0 (0)

**FIG. 1.**
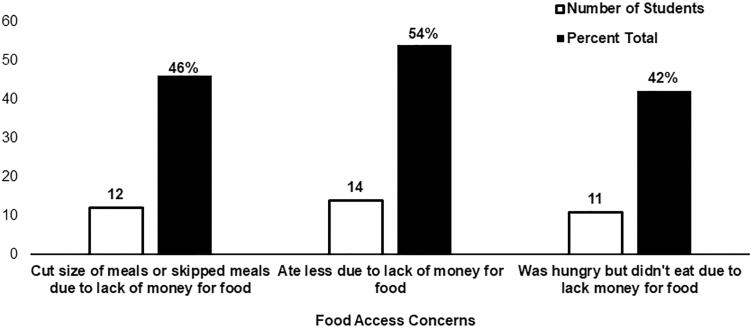
Student reported food access over the past 12 months (*n*=26).

Figure 1 shows quantitative results related to food insecurity. Over 50% of participants reported eating less due to not having money for food, and over a third cut the size of meals and skipped meals or went hungry because they lacked money for food. Fifty-three percent said that this happened some months but not every month, and 40% said that this happened almost every month over the past 12 months.

Qualitative analysis revealed five themes surrounding barriers to healthy eating: access, cost, preparation, health status, and shelf life. Access issues included limited options on campus, lack of transportation to affordable grocery stores, and access to cooking spaces. The majority of participants identified cost as a barrier to eating fruits and vegetables. One participant reported, “I can get a can of ravioli for 85 cents, and that's a full meal… a head of lettuce or broccoli costs $2+ and I need other ingredients to create a meal.” Participants also identified food preparation as a barrier due to the time and effort it takes to prepare food as well as lack of knowledge on how to prepare fruits, vegetables, or healthy meals. In addition to access, cost, and preparation, participants reported that their health status, including mental health, depression, self-worth, disability, gastrointestinal problems, and eating disorders, served as a barrier to healthy eating. In addition, many participants noted the short shelf life of fresh fruits and vegetables, which makes them difficult to eat before they spoil. Minor themes surrounding barriers to healthy eating include lack of knowledge in identifying healthy foods, desire to eat unhealthy foods, and ease of eating unhealthy foods.

### Body image

Table 3 presents quantitative results for behaviors related to body image. Over a third of participants followed a restricted diet to lose weight and 31% engaged in binge eating over the past 12 months. Almost half of the participants were attempting to lose weight, and 88% had altered their eating or exercise behaviors to try to change their body.

**Table 3.** Body Image Behaviors of Student Participants (*n*=26)

	N	%
Altered eating or exercise behaviors to attempt to change their body?		
Yes	23	88
No	2	7.7
Don't know	1	3.8
Attempting to lose weight?		
Yes	12	46
No	11	42
Don't know	3	12
Methods used to lose weight?		
Following a restricted diet	9	35
Using laxatives	0	0
Taking diet pills	1	3.8
Induce vomiting	3	12
Exercise	7	27
Other: skipping meals, avoiding eating, nothing currently	3	12
Engaged in binge eating in the last 12 months?		
Yes	8	31
No	14	54
Don't know	4	15

Qualitative analysis of open-ended questions revealed that the majority of participants viewed their body both positively and negatively. For example, one participant said, “I try to be positive and see a body that I love and value… on bad days I see a body that's sluggish and grotesque. One that can never pass for a woman,” indicating a pressure to conform to cisnormative body ideals. Other qualitative themes that emerged from questions on body image include undergoing surgery and/or using hormones and other techniques to improve body image, such as clothes, haircuts, tattoos, piercings, chest binding, and makeup. Another theme involved recognizing an eating disorder or using activities such as “fasting or only eating less than 500 calories a day, exercising every day for long periods of time.” Most participants reported exercising and/or changing their diets to try to improve their body image.

Qualitative analysis of barriers to a positive body image revealed a major theme regarding societal pressures and body standards enforced by media. One participant stated, “The world tells me that I'm not a real trans person unless I take hormones and get surgery… And then even if I do these things, being trans isn't exactly what the world extolls as beautiful. Also, as a POC [person of color], I don't often see people who look like me in the media. I have little positive representation or role models to look up to, so I don't know what good body image for someone like me looks like.” Additional themes that emerged regarding barriers to a positive body image include reactions from family members and peers, fluctuations in their gender identity and gender dysphoria, and the cost of gender-affirming interventions. For example, one participant stated, “Mental health and hormone treatment is too expensive, and I have no idea where to continue it here.”

### Resource utilization and needs

**FIG. 2.**
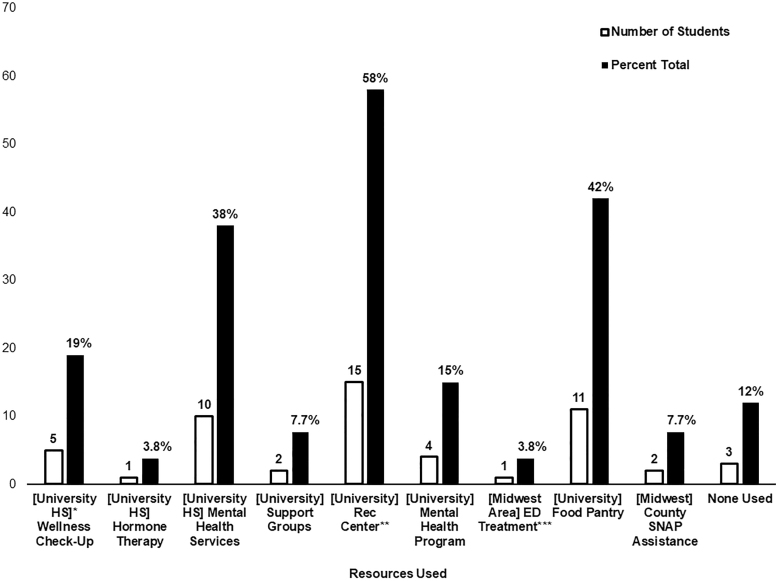
Student participant resource utilization. *University Health Service, **University Recreation and Wellness Center, ***[Midwest Area] Eating Disorder Treatment.

Quantitative data show that many of the health-related resources on the survey were underutilized by participants. However, over half of participants had utilized the campus recreational center, and over a third had utilized campus mental health services and the campus food pantry (Fig. 2). Qualitative analysis regarding nutrition resource needs revealed three main themes: participants need more free/low-cost food access resources, there is a need for more healthy food options, and there is a need for transgender/GNC nutrition counseling and education resources. Participants indicated that they enjoyed using the campus food pantry but wished it was larger and happened more frequently. Participants asked for healthier and cheaper meal plans, and identified the need to increase healthy food options on campus. They also requested nutrition counseling on applying for benefits, eating disorder recovery, and general education. Minor themes include the need for workshops or events focusing on body image, affordable nutrition, healthy meal planning, and food preservation.

Another minor theme that emerged from qualitative analysis is the importance of tailoring the resources for transgender/GNC students. For example, one student stated, “[I would like to see] nutrition consultations that are specifically for people recovering from eating disorders. I have wanted to do the ones that [the University Health Service] has but I'm very worried that they wouldn't be sensitive to my eating disorder and also not understand that my eating disorder stems from gender dysphoria and that comes with its own unique set of challenges that I need to recover from.”

Qualitative results regarding the participants' top 3 health concerns that could benefit from more resources on campus identified mental health, health care access, and nutrition and physical activity as the top concerns. Within the theme of nutrition, participants specifically identified food insecurity, healthy eating, and accessibility of treatment/diagnosis for eating disorders. Participants identified exercise as a top health concern, including transgender/GNC inclusive exercise facilities, accessible bathrooms and locker rooms, and physical disability accommodation. One participant repeatedly stated the need to consider other factors that affect their health and nutrition such as employment discrimination and lack of affordable housing.

When asked about potential nutrition interventions or programming for transgender/GNC students, qualitative analysis revealed mixed results regarding delivery format. Thirty-one percent of participants preferred one-on-one counseling, whereas 58% preferred web-based formats due to social anxiety, ease, and accessibility. Twenty-three percent of participants preferred community classes and support groups for hands-on learning and to meet other transgender/GNC students. Overall, 42% of participants were open to more than one delivery format. In addition, participants emphasized that they would want the program to be run by transgender/GNC people and be as inclusive as possible.

## Discussion

The primary focus of this study was to identify the nutrition-related needs and barriers to health among transgender/GNC students at a large public Midwestern university. The secondary focus of this study was to identify potential interventions that could be introduced on campus to improve the health and nutrition status of transgender/GNC students. Based on the results, many intervention delivery formats should be used to try to reach as many transgender/GNC students as possible. Interventions should include transgender/GNC people on the intervention team and should take place in a space where these students feel safe and comfortable. Results illuminated the underutilization of existing nutrition and health resources. Students may be unaware of these resources, or these resources may not be perceived as inclusive. Therefore, interventions should include information on available resources, and resources should be analyzed for cultural appropriateness.

Students identified cost as a barrier to nutritious foods. Taken with the rates of unemployment, annual income <$10,000, and lack of family financial support observed here (Table 1), results suggest the need for interventions to address financial burdens in this student community. Free or low-cost interventions on campus are one way to address these barriers.

Body image was identified as another intervention target. This may include supporting healthy weight management activities or focusing on nourishment and body self-acceptance. Such workshops would ideally be led by transgender/GNC facilitators, as students reported seeking relatable role models. In addition, interventions could target eating disorder prevention and treatment specifically for transgender/GNC students. This is an important issue to address, given that 42% of participants reported having an eating disorder and results highlight the need for treatment programs to be inclusive and appropriate.

Given the results of this study, general nutrition interventions should be focused on increasing consumption of fruits, vegetables, and whole grains, since these foods were consumed below recommendations. Interventions should also address the high rates of food insecurity experienced by this population by including support for food assistance enrollment, increasing access to and frequency of food pantries, or increasing the variety of financially and physically accessible healthy food options on campus. Other interventions should incorporate nutrition education on topics identified as areas of need, such as eating healthy on a budget and food preservation. As these concerns may be applicable to all students,^25,26^ consideration must be given to expanding existing programs to welcome transgender/GNC students.

Overall, study findings align with previous research regarding food insecurity, body image, and disordered eating, which are issues of concern among college students in general.^3,4^ As depicted in Table 4, this study showed significantly higher rates of food insecurity among transgender/GNC students compared with food insecurity rates among LGBTQ respondents across the Midwest region.^4^ Furthermore, transgender/GNC students in this study reported significantly higher rates of food insecurity compared with the general student population at the same university, including experiencing a food shortage and lacking money to buy more food.^27^

**Table 4.** Comparisons of the Study Sample with the General Student Population and Regional Lesbian, Gay, Bisexual, Transgender, Queer Populations

	Current study participants, N=26	Midwest LGBTQ population,^a^ N=2219	General student population at the same university,^b^ N=2023	Chi-square or Fisher's exact results (α=0.05), χ^2^* (*p)
Food insecurity				
Cut the size of meals or skipped meals due to lack of money for food	46%	24.5%	—	6.45 (0.011)
Ate less due to lack of money for food	54%	29.1%	—	7.57 (0.005)
Was hungry but didn't eat due to lack of money for food	42%	24%	—	4.68 (0.03)
Answered often to sometimes true to: the food I bought didn't last and I didn't have money to buy more	50%	—	10.2%	42.63 (<0.0001)
Dietary intake				
Zero fruits and vegetables consumed per day	15%	—	0.6%	<0.0001^c^
Body image behaviors				
Binge eating	31%	—	19%	2.30 (0.128)
Induced vomiting	12%	—	1.1%	0.0035^c^
Restricted diet	35%	—	61.7%	7.93 (0.0048)
Laxatives	0%	—	0.9%	1^c^
Exercise to lose weight	27%	—	88%	85.83 (<0.0001)
Diet pills	3.8%	—	3%	0.55^c^

^a^ These data come from: Flunker.^4^

^b^ These data come from: [University] Health Service.^27^

^c^ Fisher's exact tests were used to calculate statistical significance of variables with frequencies of five or less.

LGBTQ, lesbian, gay, bisexual, transgender, queer.

Compared with the general student population at the same university (Table 4), the transgender/GNC students in this study had significantly higher rates of not consuming fruits and vegetables daily.^27^ In addition, the transgender/GNC students in this study had nearly double the rates of binge eating compared with the general student population, although these results were not found to be statistically significant. Transgender/GNC students in this study also had significantly higher rates of inducing vomiting compared with the general student population at the same university.^27^ These findings fall in line with previous research noting higher rates of eating disorder behaviors among transgender university students.^7^ However, it is important to note the limitations of the comparisons made in Table 4 due to variations in data collection procedures and sample sizes.

### Limitations and strengths

Limitations of this study include a small sample size derived from convenience sampling. Eighty-five percent of participants identified as white and 96% were assigned female at birth. Due to resource limitations that precluded translation or presentation of study materials into multiple languages, all participants had to read and speak English to take part in the study. These factors may inhibit the generalizability of study results to other transgender/GNC populations. In addition, this study was designed as a needs assessment and lacks a formal comparison group. Furthermore, the survey was lengthy, which may have resulted in participant burden and habituation bias. Data collected were self-reported, which may be subject to social desirability bias, under-reporting, and recall bias. Member checking was not completed for qualitative analysis; however, data were captured in writing or recorded interview, which reduces errors in data collection that may arise from interviewer note-taking. In addition, data were not linked to participants with a study identifier; therefore, it was not possible to return to participants to verify that their responses were characterized adequately. Finally, this study may have benefited from measures being pretested and pilot tested since a few participants, especially those with eating disorders, felt uncomfortable answering some of the body image questions.

Strengths of this study include the novel focus on nutrition-related needs and barriers of transgender/GNC university students. Other strengths of this study include the use of qualitative methods to allow participants to elaborate their needs and ideas. In addition, this study is hypothesis generating, and may serve as a starting point for future studies and public health interventions.

## Conclusion

Despite its limitations, this study identified areas that would benefit from public health programming to improve nutrition and health of transgender/GNC students. Many of these areas impact one another, and all play a role in the academic success and well-being of these students. By addressing disparities among college students, we help ensure all students are given the opportunity to be successful, given that obtaining a degree in higher education has been linked to improved health and socioeconomic opportunity.^4,10^

## Abbreviations Used

CSS: College Student Health Survey
GNC: gender nonconforming
IRB: Institutional Review Board
LGBTQ: lesbian, gay, bisexual, transgender, queer
VHS: Voices of Health
